# In Silico Study of the Potential of Brazilein Sappan Wood as a Beta-Lactamase Inhibitor against Extended-Spectrum Beta-Lactamase-Encoding Genes

**DOI:** 10.21315/mjms2024.31.3.7

**Published:** 2024-06-27

**Authors:** Dwi Krihariyani, Evy Diah Woelansari, Edy Haryanto, Retno Sasongkowati, Anik Handayati, Sri Sulami Endah Astuti

**Affiliations:** Department of Medical Laboratory Technology, Politeknik Kesehatan Kementerian Kesehatan Surabaya, East Java, Indonesia

**Keywords:** brazilein, clavulanic acid, extended-spectrum beta-lactamase coding gene, in silico test

## Abstract

**Background:**

Infectious illnesses are a serious health concern in Indonesia. Widespread use of self-medication by the community increases the risk of developing multi-drug resistant (MDR) bacteria. This study assessed the potential of sappan wood as an inhibitor of extended-spectrum beta-lactamase (ESBL) encoded by blaSHV, blaTEM and blaCTX-M genes.

**Method:**

In silico testing was conducted to develop an effective and economical starting strategy. Thereby, this study significantly advances the development of novel treatments to combat antibiotic resistance. Using clavulanic acid as the benchmark medicine, the potency of the beta-lactamase inhibitor brazilein was predicted. Using the Molegro Virtual Docker computer tool, docking was performed to estimate the chemical and physical properties of the compounds, as well as the biological activity of brazilein toward the required receptor. The receptors used were SHV-1 beta-lactamase, PDB code: 2H0T; TEM-1 beta-lactamase, PDB code: 4OQG and CTX-M-14 beta-lactamase, PDB code: 6VHS. Data analysis was performed by comparing the binding energies of the docking results between the ligands and the target receptor. The more stable the bond that formed between the ligand and the target receptor, the lower the bond energy.

**Results:**

The in silico test results on the blaSHV gene were as follows: binding energy of ligand MA4_400[A] = −100.699, brazilein = −82.206, clavulanic acid = −79.3704; in the blaTEM gene: ligand bond energy 2UL_301[B] = −107.681, brazilein = −82.0296, clavulanic acid = −103.3; in the blaCTX-M gene: X57_301[A] ligand bond energy = −86.6197, and brazilein = −88.1586, clavulanic acid = −101.933.

**Conclusion:**

The findings of this study demonstrate the significant potential of brazilein sappan wood to block the beta-lactamase activity of blaCTX-M.

## Introduction

Infectious diseases are a major problem (1, 2). Antibiotic resistance, particularly that caused by multidrug-resistant (MDR) microbes, has emerged as a significant obstacle in treating illnesses (3, 4). The widespread practice of community self-medication, which involves incorrect use of antibiotics without adequate medical supervision, is one of the factors causing an increase in antibiotic resistance (5). Beta-lactam antibiotic resistance in bacteria, particularly that caused by beta-lactamases (6). This poses a serious public health (7). Combination therapy using antibiotics and medications that function as beta-lactamase inhibitors has recently been proven to be the most effective method for treating MDR bacterial infections (8). The increase in MDR bacterial infections has sparked efforts to create new inhibitory drug combinations and expand the materials available for their production, such as sappan wood (*Caesalpinia sappan* L.), which may assist in combating beta-lactamase-caused antibiotic resistance.

Numerous studies have investigated the use of these inhibitors. Based on Yahav et al. (9), the discovery of a combination of beta-lactam-lactamase inhibitors (BLBLIs) shows potential as a novel therapeutic option for carbapenem-resistant Enterobacterales, *Pseudomonas aeruginosa, Acinetobacter baumannii* and other bacteria, including mycobacteria. However, the use of this substitute is constrained by the high cost of BLBLIs. Mojica et al. (10) studied the creation of novel boronate compounds that are effective against the majority of metallo-β-lactamases B1 and the work was similar. Despite being in the early stages, the development of boronate-based inhibitors appears promising. However, there are restrictions on the supply of boronates, and a lack of knowledge regarding their inhibitory mechanisms and ranges of activity. Stewart et al. (11) reported that a novel combination of cefpodoxime/ETX0282 and ceftibuten/VNRX7145 was created. This new combination also demonstrates excellent efficacy against extended-spectrum beta-lactamase (ESBL) producers. Limited quantities of ETX0282 and VNRX7145 are available; entasis therapeutics continues to work on these and is awaiting clinical trials.

The use of medicinal herbs, which are widely available in Indonesia, is a dependable alternative because the materials for creating beta-lactamase inhibitor medications that have been created still have limits. In vitro and in vivo studies by Lee et al. (12) demonstrate that the flavonoid compounds in rhamnetin significantly reduce inflammation in lipopolysaccharide-stimulated mouse macrophages, carbapenem-resistant *A. baumannii* (CRAB) and *Escherichia coli* by preventing the release of nitric oxide and interleukin-6. Similar work was done in 2021 by Song et al. (13), who used minimum inhibitory concentrations (MICs) and plant flavonoids as antibacterials to combat MDR bacteria. The findings of the study indicate that natural plant products can be a useful source and should be investigated further in order to combat bacteria that are resistant to antibiotics. There are various benefits to the development of beta-lactamase inhibitors from natural materials such as sappan wood. First, medicinal plants contain several active substances that can work in concert with one another and block the activity of the beta-lactamase enzyme through various modes of action. Second, employing natural substances can help sustain the diversity of natural resources by reducing reliance on synthetic antibiotics.

Plant-derived flavonoid compounds act against MDR bacteria (14); therefore, further studies are required to determine the advantages of these flavonoid compounds as beta-lactamase inhibitors (15). Five flavonoid-related active substances have been reported in sappan wood. Based on empirical evidence, sappan wood is a type of medication (16). Brazilein is a major secondary metabolite in sappan wood. Thus, it is possible to develop a beta-lactamase inhibitor from sappan wood bran. Conduct an in silico test with the use of a computer utilising the Molegro Virtual Docker programme is important to assess the potential of brazilein sappan wood as a beta-lactamase inhibitor against ESBL expressing genes (blaSHV, blaTEM and blaCTX-M) (17). Using a molecular model, an in silico test was utilised to predict the physicochemical properties and biological activity (18, 19). One benefit of in silico testing is that it is rapid, simple, inexpensive, safe and free of chemical waste (18, 19).

Using clavulanic acid as a benchmark, the effectiveness of brazilein sappan wood as a beta-lactamase inhibitor was evaluated (20). When docking on the protein data bank, ligands or compounds that have demonstrated strong biological activity and can attach to the desired biological target include the crystal structure of the M69V E166A double mutant of SHV-1 beta-lactamase complexed to clavulanic acid (PDB ID: 2H0T), crystal structure of TEM-1 beta-lactamase in complex with boron-based inhibitor EC25 (PDB ID: 4OQG) and crystal structure of CTX-M-14 in complex with beta-lactamase inhibitor ETX1317 (PDB ID: 6VHS). Therefore, using clavulanic acid as a comparator, this study aimed to evaluate the potential of brazilein Secang wood as a beta-lactamase inhibitor against ESBL-expressing genes (blaSHV, blaTEM and blaCTX-M). The discovery of beta-lactamase inhibitors from natural materials, particularly brazilein sappan wood, is a contribution of this study. These inhibitors assist in minimising the reliance on manufactured antibiotics and exploit a variety of existing natural resources. In silico testing has several benefits including safety, elimination of chemical waste, cost-effectiveness and time savings (21). The effectiveness of brazilein as a beta-lactamase inhibitor can also be rapidly and accurately predicted using this in silico test (22).

## Methods

### Download of the Target Protein (blaSHV, blaTEM, blaCTX-M)

The RCSB Protein Data Bank (sPDB) was used to download target proteins. Research was carried out using the proper beta-lactamase receptors to discover possible protein targets of the blaSHV, blaTEM and blaCTX-M genes as targets for the development of antibacterial therapy. The ligand tested for blaSHV was MA4_400[A] and the receptor employed was SHV-1 beta-lactamase with PDB code 2H0T. The TEM-1 beta-lactamase receptor with PDB code 4OQG was used as the blaTEM gene and the ligand under study was 2UL_301[B]. CTX-M-14 beta-lactamase is the receptor used by the blaCTX-M gene and X57_301[A] is the ligand that was tested.

### Prediction of Activity and Amino Acid Analysis

ChemDraw Professional 16.0 was used to create the two-dimensional (2D) structures of the compounds to be docked, followed by conversion to 3D using Chem3D 16.0 and selection of the most stable conformation. The minimum energy was measured and stored as mol2 {SYBYL2(*. mol2)}. The docking process was then performed using the Molegro Virtual Docker 5 computer programme against the SHV-1 beta-lactamase receptor target, PDB code: 2H0T; TEM-1 beta-lactamase, PDB code: 4OQG; and CTX-M-14 beta-lactamase, PDB code: 6VHS. It is possible to anticipate the potential of brazilein sappan wood as a beta-lactamase inhibitor against ESBL-expressing genes (blaSHV, blaTEM and blaCTX-M) from the data obtained, which are Rerank Score (RS) values that represent the energy required in the process of ligand-receptor contact (23, 24).

## Results

**Figure 1:**
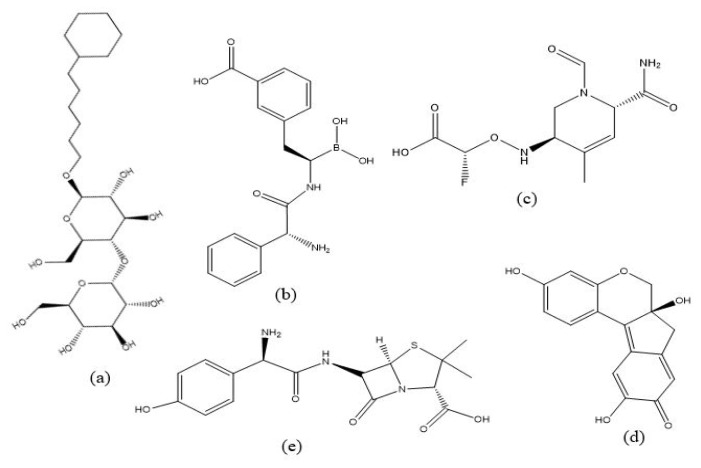
2D structure (a) MA4 ligand, (b) 2UL ligand, (c) ligand X57, (d) brazilein and (e) clavulanic acid

**Figure 2:**
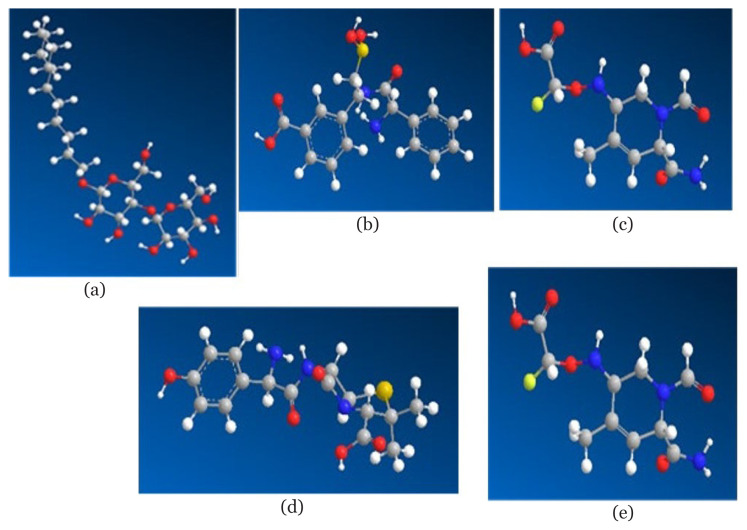
Shape of the 3-D structure illustrated using SYBYL2. (a) MA4 ligands, (b) 2UL ligand, (c) ligand X57, (d) brazilein and (e) clavulanic acid

Figure 1 shows the results of producing a 2D structure using ChemDraw Professional 16.0. Chem3D 16.0 is then used to convert the 2D structure into a 3D structure. Figure 2 shows the 3D framework used during the docking stages.

The interactions of many important beta-lactamase amino acid residues, including SHV-1, TEM-1, and CTX-M-14, with three distinct ligands, including MA4_400[A], 2UL_301[B], X57_301[A], brazilein and clavulanic acid, are shown in Tables 1, 2 and 3. In these table, steric effects and H-bonding are the two main methods by which receptors and ligands interact.

The outcomes of beta-lactamase receptor re-docking using the Molegro Virtual Docker 5 computer programme are displayed in Table 4 and include SHV-1 (PDB code: 2H0T), TEM-1 (PDB code: 4OQG) and CTX-M-14 (PDB code: 6VHS). The outcomes of this redocking provided a better understanding of the interactions between various ligands and each receptor. The design and improvement of compounds with the potential to function as beta-lactamase inhibitors, which are crucial in the fight against antibiotic resistance and the creation of more potent antibacterial treatments, could greatly benefit from this knowledge.[Table t4-07mjms3103_oa]

## Discussion

The target molecular receptor structures used were the SHV-1 beta-lactamase receptor, PDB code: 2H0T, ligand MA4_400[A]; TEM-1 beta-lactamase receptor, code PDB; 4OQG, 2UL_301[B] ligand and CTX-M-14 beta-lactamase receptor, PDB code: 6VHS, ligand X57_301[A]. The blaSHV, blaTEM and blaCTX-M genes, which encode model proteins similar to the ESBL-coding genes, are the three target receptors downloaded from the PDB. ESBL, which may hydrolyse penicillins, cephalosporins and monobactams, are encoded by the TEM, SHV and CTX-M genes (25, 26). The ESBL gene, encoded by a plasmid of the TEM (Temoniera) derivative family, sulfhydryl variable (SHV) and oxacillinase (OXA), is the source of traditional enzymes (27).

The MA4_400[A], 2UL_301[B] and X57_301[A] ligands were selected to identify the chemical-physical properties of the molecule, as well as because they have demonstrated good biological activity and may bind to the intended biological target (receptor) during the docking procedure. To design the minimal structural features necessary for further drug development, a computer programme can be used to search for groups that are responsible for activity (pharmacophores) and groups that can reduce activity, as well as the lipophilic, electronic and steric/geometric properties of these groups (26, 28).

Drug delivery to receptors is significantly influenced by their physical and chemical characteristics. Drug distribution and absorption are influenced by the physicochemical characteristics of the drug (lipophilic and electronic), resulting in high drug concentration at the receptor. Only drugs with a high degree of specificity can interact with biological receptors and induce activity. The particular orientation of the molecules on the receptor surface is supported by the physicochemical (electronic and steric) characteristics of the medicines (30).

When receptors and ligands interact, the amino acid residues produced often interact with each other through lipophilic/hydrophobic bonds and steric effects. This interaction involves a considerable number of amino acid residues, suggesting that the ligand has a high probability of binding to the target protein. The steric effect refers to the effects of physical space and obstruction caused by the shape and size of the ligand, whereas the lipophilic/hydrophobic link produces an attraction between the hydrophobic component of the ligand and amino acid residues in the receptor, which have similar qualities. In the context of biomedical and pharmaceutical research, this knowledge is crucial for creating and refining compounds that have the potential to act as binding agents for therapies or inhibit particular target proteins (29, 30).

The findings of this investigation provide a summary of the mechanism by which brazilein, a secondary metabolite of the flavonoid class, inhibits beta-lactam synthesis (31). There are numerous proposed processes for how flavonoids work as beta-lactamase inhibitors, although they have not all been thoroughly elucidated: i) suppresses the synthesis of enzymes (32). The manufacture of beta-lactamases can be stopped by flavonoids at the transcriptional or translational level by reducing the synthesis of the enzymes required to stop beta-lactamase activity, ii) blocking enzyme activity (33). By binding to beta-lactamases either reversibly or irreversibly, altering the enzyme’s structure or blocking the chemical processes that the enzyme needs to function, flavonoids can directly interact with beta-lactamases and decrease their catalytic activities (34), iii) increases the effect of antibiotics. By altering membrane permeability, which enables antibiotics to enter bacterial cells more easily and impede the growth of MDR pathogens, flavonoids can enhance the effectiveness of beta-lactam antibiotics (35).

Although it cannot fully replace genuine clinical and biological testing, in silico testing can offer useful initial insights into the process of drug creation and evaluation of biological activity. In silico testing is used as a preliminary test before in vitro and in vivo testing and remains a vital stage in the discovery of novel medications.

In silico studies can shed light on the potential of sappan wood as a source of novel medications that can combat antibiotic resistance caused by beta-lactamases encoded by the blaSHV, blaTEM and blaCTX-M genes. Techniques for creating potent inhibitors can also be developed.

## Conclusion

In this investigation, the ESBL gene encoded by blaSHV, blaTEM and blaCTX-M will be tested for its potential as a beta-lactamase inhibitor. The lower the bond energy of the ligand with the target receptor, the more stable the bond. The outcomes demonstrated the potential of brazilein sappan wood to block the beta-lactamase activity of blaCTX-M. In vivo tests using experimental animals, bioactivity tests, activity structure analyses, structure optimisation, in vitro tests, toxicity and pharmacokinetic studies and collaboration with other researchers in relevant scientific fields can help advance further research.

## Figures and Tables

**Table 1:** Amino acid residues implicated in the H-bond and steric bond interactions between SHV-1 beta-lactamase and its ligands

Receptor	Ligand	Residue amount	H-bonding and residues of amino acids	Residue amount	Residues of amino acids and steric bonds
SHV-1 beta-lactamase	MA4_400[A]	1	Val 224	4	Val 224; Pro 226; Ile 287; Glu 288
	brazilein	3	Val 224; Leu 225; Pro 226	4	Val 224; Leu 225; Pro 226; Ser 223
	clavulanic acid	0	–	5	Pro 226; Il 221; Leu 225; Val 224; Ala 280

**Table 2:** Amino acid residues implicated in the H-bond and steric bond interactions between TEM-1 beta-lactamase and its ligands

Receptor	Ligand	Residue amount	H-bonding and residues of amino acids	Residue amount	Residues of amino acids and steric bonds
TEM-1 beta-lactamase	2UL_301[B]	7	Asn132(B); Asn170(B); Glu166(B); Ala 237(B); Ser 130(B); Ser 235(B); Arg 244(B)	12	Asn 132(B); Asn 170(B); Glu 104(B); Glu 197(B); Glu 166(B); Tyr 105(B); Ala 237(B); Gly 236(B); Ser 130(B); Ser 70(B); Ser 235(B); Arg 244(B)
	brazilein	3	Ser 235(B); Val 216(B); Ser 70(B)	9	Ala 237(B); Met 272(B); Tyr105(B); Ser 70(B); Val 216(B); Arg 244(B); Pro 219(B); Ala 217(B); Ser 235(B)
	clavulanic acid	4	Val 108(B); Asn 132(B); Ser 130(B); Lys 73(B)	13	Ala 134(B); Ser 106(B); Glu 104(B); Thr 133(B); Thr 109(B); Pro 107(B); Asp 131(B); Val 108(B); Tyr 105(B); Ser 130(B); Lys 73(B); Ser 70(B); Asn 132(B)

**Table 3:** Amino acid residues implicated in the H-bond and steric bond interactions between CTX-M-14 beta-lactamase and its ligands

Receptor	Ligand	Residue amount	H-bonding and residues of amino acids	Residue amount	Residues of amino acids and steric bonds
CTX-M-14 beta-lactamase	X57_301[A]	5	Thr 235(A); Ser 237(A); Ser 70(A); Asn 132(A); Asn 104(A)	8	Thr 235(A); Ser 237(A); Ser 130(A); Gly 236(A); Ser 70(A); Asn 132(A); Asn 170(A); Asn 104(A)
	brazilein	3	Ser 237(A); Thr 227 (B); Asn 132(A)	8	Ser 237(A); Gly 238(A); Asn 104(A) Asn 132(A); Ser 130(A); Lys 73(A); Ser 70(A); Thr 227(B)
	clavulanic acid	3	Ser 130(A); Ser 237(A); Asn 170(A)	12	Tyr 105(A); Leu 169(A); Ser 130(A); Ser 70(A); Ser 237(A); Gly 238(A); Asn 170(A); Pro 167(A); Thr 168(A); Thr 171(A); Asp 240(A); Ala 172(A)

**Table 4 t4-07mjms3103_oa:** Redocking results using the Molegro virtual docking programme

ESBL encoding genes	Ligand	Rerank score
blaSHV	MA4_400[A]	−100.699
	brazilein	−82.206
	clavulanic acid	−79.3704
blaTEM	2UL_301[B]	−107.681
	brazilein	−82.0296
	clavulanic acid	−103.3
blaCTX-M	X57_301[A]	−86.6197
	brazilein	−88.1586
	clavulanic acid	−101.933
